# Phase 2 study of perioperative chemotherapy with SOX and surgery for stage III colorectal cancer (SOS3 study)

**DOI:** 10.1038/s41598-019-53096-3

**Published:** 2019-11-12

**Authors:** Naoya Aisu, Yoichiro Yoshida, Akira Komono, Ryohei Sakamoto, Daibo Kojima, Suguru Hasegawa

**Affiliations:** Department of Gastroenterological Surgery, Fukuoka University School of Medicine, Nanakuma 7-45-1, Jonan-ku, Fukuoka 814-0180 Japan

**Keywords:** Colon cancer, Surgical oncology

## Abstract

This phase 2 study evaluated the safety and efficacy of perioperative chemotherapy with S-1 plus oxaliplatin (SOX) for stage III colorectal cancer (CRC). Patients with stage III CRC received surgery after neoadjuvant chemotherapy (NAC; SOX 4 cycles) and adjuvant chemotherapy (AC; SOX 4 cycles). The primary endpoints were response rate and safety. We enrolled 30 patients. Their median age was 62 years (range: 43–87 years); 53% were women. They received a median of 4 cycles (range: 1–4) of NAC and a median 4 cycles (range: 0–4) of AC. Five patients interrupted NAC treatment because of toxicity (grade 3 diarrhoea [*n* = 1], grade 3 ileus [*n* = 1], and grade 3–4 thrombocytopenia [*n* = 3]). Patients’ responses were complete responses: *n* = 2 (6.6%), partial responses: *n* = 21 (70%), stable disease: *n* = 6 (20.0%), and progressive disease: *n* = 1 (3.3%; response rate: 73.3%). Curative resection was performed in 29 patients. No patients showed anastomotic leakage. Five-year overall survival and disease-free survival were 83.3% and 76.7%, respectively (median follow-up time: 48 months). NAC using SOX regimen is safe and effective, and may lead to reduced local recurrence and distant metastasis. Long-term outcomes are awaited to evaluate further the efficacy of this strategy (UMIN000006790).

## Introduction

Colorectal cancer (CRC) is second-most common cancer in the world. Among patients with resectable CRC, the 5-year survival rate has improved to more than 80%^1^. However, the survival rate of patients with stage III CRC is still not satisfactory. To improve outcomes, postoperative adjuvant chemotherapy (AC) is usually recommended to suppress distant micrometastasis; it has shown a 63–74% improvement in 5-year survival over surgery alone^2^. To improve outcomes further, some researchers investigated the effectiveness of administering cytotoxic therapy as neoadjuvant chemotherapy (NAC)^2^. Potentially, NAC can provide *in vivo* therapeutic responses, tumour downstaging, and early treatment for micrometastatic disease. However, NAC for CRC has several potential drawbacks, such as possible disease progression, surgery postponement due to adverse event (AEs), and increased postoperative complications. Although NAC is promising, data demonstrating its safety and efficacy are still scarce.

FOLFOX and CAPOX has been used in clinical trials of NAC for stage III CRC^3–6^. Although CAPOX and SOX reportedly have equivalent effects in metastatic CRC^7,8^, clinical trials that use SOX therapy as NAC have not been reported for stage III CRC. AEs are thought to differ between SOX and CAPOX, as S-1 and capecitabine have different metabolic pathways^9^. AEs associated with NAC over a limited administration period are unknown. This trial evaluated the efficacy and safety of NAC for clinical lymph-node-positive CRC. Here, we present the short-term results of this trial, with respect to response rates, toxicities and surgical complications.

## Results

### Patient characteristics

We enrolled 30 patients who were treated between March 2012 and September 2016. Their median age was 62 years (range: 43–87); 53% were women. Table 1 summarizes their baseline characteristics. Their median body mass index was 21.6 kg/m^2^. Their ECOG-PS scores were 0: 40%, 1: 60%. The median length of the primary tumour at the baseline was 50 mm (range: 20–102 mm); distribution was cT2: *n* = 1, cT3: *n* = 12, and cT4: *n* = 17 (cT4a: *n* = 12, cT4b: *n* = 5).

**Table 1:** Patients’ baseline characteristics.

		N = 30 (%)
Age(years)	median(range)	62 (43–87)
Gender	Male	14(47)
	Female	16(53)
Primary site	Colon	9(30)
	Rectum	21(70)
Primary tumour size	median(range)	50 (20–103)
ECOG performance status	0	12(40)
	1	18(60)
BMI (kg/m^2^)	<20	10(33)
	20<, <24	14(47)
	>24	6(20)
Clinical T stage	T2	1(3)
	T3	12(40)
	T4a	12(40)
	T4b	5(17)
Clinical N stage	N1	16(53)
	N2	14(47)
Histological grade	well	23(77)
	moderately	6(20)
	poorly	1(3)

BMI: Body Mass Index; ECOG: Eastern Cooperative Oncology Group.

### Treatment compliance and toxicity of NAC and AC

Of the 30 patients enrolled in this study, 25 patients completed NAC. The patients received median 4 (1–4) cycles of NAC and median 4 (0–4) cycles of AC. Their total median dose of oxaliplatin was 635 (122–1040) mg/m^2^ (NAC; 494 [122–520] mg/m^2^, AC; 230 [0–520] mg/m^2^). Five patients interrupted NAC due to AEs (grade 3 diarrhoea [*n* = 1], grade 3 ileus [*n* = 1], grade 3–4 thrombocytopenia [*n* = 3]). AC was administered to 21 patients (17: SOX: *n* = 17, S-1 only: *n* = 4). Eight patients did not received AC.

AEs during NAC are shown in Table 2. The most common AEs were thrombocytopenia (*n* = 16, 53%) and neutropenia (*n* = 11, 37%) for haematological events; and peripheral neuropathy (*n* = 25, 83%) and fatigue (*n* = 18, 60%) for non-haematological events. Two patients had grade 4 AEs (diarrhoea and thrombocytopenia).

**Table 2:** Adverse events during NAC.

	All grades, N (%)	Grade3 or 4, N (%)
Haematological		
Thrombocytopenia	16(53)	4(13)
Neutropenia	11(37)	3(10)
Non-haematological		
Fatigue	18(60)	1(3)
Diarrhoea	5(17)	2(7)
Nausea	12(40)	0
Bleeding	1(3)	1(3)
Hand-foot syndrome	9(30)	0
Peripheral neuropathy	25(83)	0
Hypersensitivity	1(3)	0

### Effect of chemotherapy

**Figure 1:**
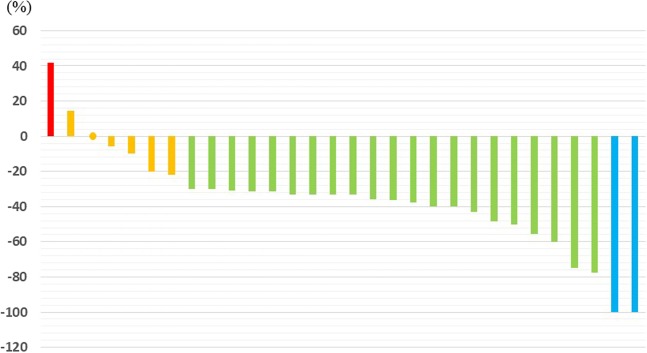
Waterfall plot shows changes in primary tumour diameters observed at the end of NAC compared with baseline (red: PD, orange: SD, green: PR, blue: CR).

The response rate to NAC in this study, according to RECIST ver.1.1, was 76.6% (complete response: 6.6%; partial response: 70.0%; stable disease: 20.0%; and progressive disease: 3.3%). The disease control rate was 96.7%. One patient who was not responsive was shown to have para-aortic lymph node (LN) metastasis on computed tomography (CT) after 4 cycles of SOX. The median tumour shrinkage rate was 33% (range: −42–100%) after NAC; 23 of the 30 patients achieved a > 30% reduction in tumour size. The waterfall plot (Fig. 1) shows the rate of change in primary tumour diameters at the end of NAC compared with baseline.

### Surgical outcomes

Of the 30 patients who received NAC, 29 underwent curative surgery (Table 3). One patient received a pancreaticoduodenectomy in addition to right hemicolectomy. Procedures were laparoscopic: 55.2%, and open: 44.8%. Median surgical time was 335 (72–729) minutes. Median estimated blood loss was 129 (0–2210) g. Twelve patients (41.3%) suffered postoperative complications; 2 patients, respectively, experienced pancreatic fistula and lower-leg compartment syndrome (Clavien–Dindo grade ≥ 3). Although no anastomotic leakage was observed, 13 patients received ileostomies or colostomies.

**Table 3:** Surgical results (*N* = 29).

Surgical results		
Bleeding (gram median)	129(0–2210)	
operative time (min median)	335(72–729)	
	**n**	**%**
Operative procedures		
colectomy	3	10.3
hemicolectomy	4	13.8
Low anterior resection	12	41.4
Intersphincteric resection	2	6.9
Hartmann	1	3.4
Abdominoperineal resection	7	24.2
Lateral lymph node dissection		
Yes	2	6.9
No	0	0
Laparoscopic		
Yes	13	44.8
No	16	55.2
Postoperative complication		
wound infection	4	13.8
pancreatic fistula	1	3.4
Bowel obstruction	2	6.9
Urinary infection	1	3.4
pelvic sepsis	1	3.4
compartment syndrome of lower legs	1	3.4

### Pathological assessment

Three (10.3%) of the 29 patients who underwent resections showed pCR, and 12 (41.4%) exhibited good tumour regression (grade 2/3). One patient’s primary tumour disappeared but his LN metastasis remained. Table 4 shows the relationships between clinical stage before chemotherapy and pathological stage. After NAC, the frequency of T downstaging was 69.0% (20/29) and disappearance of LN metastasis was 65.5% (19/29).

**Table 4:** Relationship between clinical stage before chemotherapy and pathological stage.

	ypT0	ypT1	ypT2	ypT3	ypT4a	ypT4b	ypN+
cT2(N = 1)	1	-	-	-	-	-	
cT3(N = 12)	1	2	6	2	1	-	3
cT4a(N = 12)	2	-	3	3	5	-	7
cT4b(N = 4)	-	-	-	-	2	2	
cN + (N = 29)	4	2	9	5	7	2	10

c: clinical assessment data; yp: pathological data after systemic or radiation therapy (either prior to surgery or as a primary treatment).

### Prognosis

**Figure 2:**
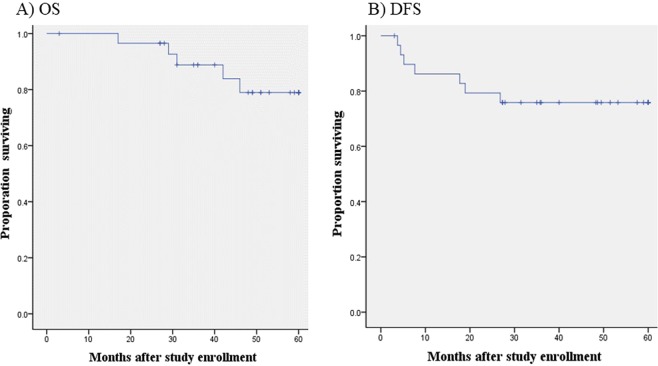
(**A**) Overall survival, and (**B**) disease-free survival for the 30 patients eligible for this study. Their 3-year overall survival and progression-free survival rates were 83.3% and 76.7%, respectively.

Five-year OS and DFS were 83.3% and 76.7%, respectively (median follow-up time 48 months); OS and DFS curves are shown in Fig. 2. Of the 29 patients who underwent R0 resection, 7 patients had relapsed. Lung recurrence was seen in 2 patients, liver recurrence in 1 patient, LN recurrence in 3 patients, and local recurrence in 2 patients (colon: 0, rectum: 2).

## Discussion

Whereas surgery removes obvious tumours, NAC provides the advantage of anticancer drug delivery, to treat distant micrometastases that have not been detected, potentially reducing the risk of recurrence^10–12^. Use of NAC can also help in estimating response to chemotherapy, and thus in selecting the optimal adjuvant therapy. NAC is used to reduce tumour size and render initially unresectable tumours resectable^13,14^.

This trial evaluated the efficacy and safety of SOX as NAC for patients with clinical node-positive CRC. Because port-free chemotherapy is suitable for perioperative patients, and can avoid complications associated with central venous ports^15^, the SOX regimen was chosen over the FOLFOX regimen. S-1 can lead to less hand-foot syndrome (HFS) than capecitabine^16^ and several studies have demonstrated the efficacy of SOX for metastatic CRC^17,18^, As the AVOID trial demonstrated that adding dexamethasone could help prevent oxaliplatin-induced hypersensitivity reactions^19^, we mixed dexamethasone (6.6 mg) into the oxaliplatin.

However, this is the first report of SOX therapy as a preoperative chemotherapy. Hong *et al*. had shown that patients treated with SOX had greater overall objective responses than did those treated with CAPOX, and that S-1 was more advantageous than capecitabine in reducing HFS frequency^7^, They also showed clear noninferiority of SOX and CAPOX with respect to progression-free survival. Thrombocytopenia is an important haematological AE of oxaliplatin. However, whereas 22% of patients treated with SOX reportedly suffer grade 3–4 thrombocytopenia^7^, the incidence of grade 3–4 thrombocytopenia in this study is 13.8%—lower than previous studies. This probably reflects our study’s drug-withdrawal period for surgery. AE rates in this trial differed from reported rates for CAPEOX as NAC^3–5^. SOX appears to be especially good for patients in occupations that require manual dexterity because of the low HFS incidence.

In this study, the response rate was 76.6%, and tumour control rate was 96.7%, with 29 patients undergoing curative resections. Two patients showed perioperative complications grade ≥ 3 (Clavien–Dindo classification), but these were not thought to be directly related to NAC. Overall survival and relapse-free survival were 83.3% and 76.7%, respectively (median follow-up time 48 months). These results are not inferior to those of other reported clinical trials^3–6^. SOX therapy is a better regimen for those who do not want to have a CV port or those suffering from severe HFS.

Preoperative chemoradiotherapy (CRT) combined with total TME has improved local control and sphincter preservation rates in patients with locally advanced rectal cancer^20^. The reported 3-year DFS of CRT for patients with locally advanced rectal cancer is 71.6–75.9%^21–23^, which is equivalent to the results of the present study. However, CRT has been recently shown to increase perioperative complications, such as local infections and anastomotic leakage^6^. Furthermore, irradiated patients may develop later complications of bowel, bladder, and sexual function^24^. However, NAC for CRC also has some potential disadvantages, including the increased possibility of postsurgical complications. Although NAC is theoretically promising, data to support its safety and efficacy is still inadequate.

This trial had some limitations. First, the study cohort was rather small. Second, preoperative staging is not always accurate, which could have led to overtreatment of patients who may not need NAC. To increase dose intensity, we recommend examining results of other SOX studies and setting the administration method for the phase 3 study. Despite these potential limitations, we believe this strategy is promising as NAC for stage III CRC.

## Conclusions

This research confirmed the feasibility of NAC using SOX regimen for stage III CRC. However, the results of the phase 3 trial will better indicate whether this regimen reduces local recurrence and distant metastasis rates.

## Patients and Methods

This study was a single-arm, open-label, prospective, phase 2 study to evaluate the response rate and safety of NAC with SOX therapy for stage III CRC. The study protocol was approved by the institutional review board of Fukuoka University Hospital (No. 11-056). This study is registered with UMIN-CTR, UMIN000006790 (first registration date: 28/Nov/2011). Informed consent was obtained from all patients prior to study entry. All procedures were performed in accordance with the Declaration of Helsinki.

### Eligibility criteria

All patients had pathologically confirmed adenocarcinoma of the colon and rectum without evidence of distant metastasis. The inclusion criteria for this study were stage III CRC (any T and N1–N3). The stage was assessed by colonoscopy, computed tomography (CT), or magnetic resonance imaging (MRI). The definition of LN metastasis was determined by CT or MRI as follows: colon: minimum LN diameter > 10 mm as determined by CT^25–27^; rectum: LN > 5 mm in short-axis diameter with an irregular border or mixed signal intensity as determined by MRI^28,29^. Patients had Eastern Cooperative Oncology Group Performance Status (ECOG-PS) of 0 or 1 and preserved organ function, and had provided written informed consent. Exclusion criteria included distant metastasis or synchronous tumours, history of neurologic or psychiatric disorders, cardiovascular disease, previous history of chemotherapy or pelvic radiotherapy, or another cancer diagnosis within the past 5 years.

### Treatment protocol

Patients with stage III CRC received NAC (4 cycles of SOX) before surgery and AC (4 cycles of SOX) after surgery. Four cycles of SOX were administered before surgery, because many clinical trials of CAPOX-based NAC use four cycles of treatment^3,4,30^.

#### Neoadjuvant treatment

**Figure 3:**
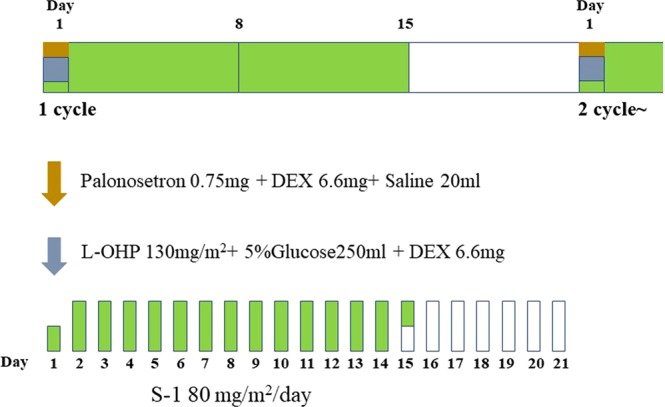
SOX regimen: S-1 (80 mg/m^2^/day) from Days 1 to 14, oxaliplatin (85 mg/m^2^) on Day 1, every 3 weeks.

The treatment schema is shown in Fig. 3. In principle, patients received 4 cycles of NAC, consisting of SOX: S-1 (80 mg/m^2^/day) from Days 1 to 14, and oxaliplatin (85 mg/m^2^) on Day 1, every 3 weeks. To prevent oxaliplatin-induced hypersensitivity reaction, dexamethasone and oxaliplatin were co-injected^19^. Toxicity was assessed according to the National Cancer Institute Common Terminology for Adverse Events, version 4.0. We used the Response Evaluation Criteria in Solid Tumors (RECIST) guidelines to determine objective responses of target lesions.

#### Surgery

After four cycles of neoadjuvant SOX therapy, tumour resectability was reassessed using CT. Patients for whom surgery was suitable underwent complete mesocolic excisions or total mesorectal excisions (TME) at 4–6 weeks after their last NAC courses. Combined resections were performed when cancer was intraoperatively suspected to have invaded other organs. Selection of an open or laparoscopic approach was at the surgeon’s discretion. Postoperative morbidities were assessed according to the Clavien–Dindo classification.

#### Adjuvant chemotherapy

Patients for whom NAC was effective (i.e., had no progressive disease) and tolerable, and who had R0 resections received postoperative AC with four cycles of SOX.

### Study end points

The primary end points of this study were objective response rate and safety. The secondary end points include NAC completion rate, relative dose intensity, incidence of AEs, postoperative complications, pathologic tumour response, and 5-year overall survival (OS) and disease-free survival (DFS). Pathologic tumour responses were graded according to the Japanese Classification of Colorectal Carcinoma^31^ as 1a: denaturation and necrosis of cancer cells in less than a third of the cancer tissue; 1b: denaturation and necrosis in less than two thirds of the cancer cells, plus fusion in more than one third of the cancer; 2: significant denaturation, necrosis, fusion, and loss in more than two thirds of the cancer; and 3: pathologic complete response (pCR) with no cancer cells observed in either the primary or regional LNs.

### Sample size and statistical analysis

This study aims to reduce the risk of recurrence of stage III CRC, using pre- and postoperative SOX therapy. Therefore, the response rate to SOX therapy in preoperative chemotherapy was regarded as the primary endpoint. In clinical trials for metastatic CRC, response rates for FOLFOX, CAPOX, and SOX were 41–49%^32–34^, 27–55%^32–35^, and 50%^36^, respectively. However, according to an integrated analysis of results of randomized controlled trials of FOLFOX and CAPOX, CAPOX therapy is inferior to FOLFOX therapy in response rates (odds ratio: 0.74)^37^. The planned sample size was determined by defining expected and threshold response rates as 50% and 30%, respectively. By setting alpha and beta errors at 0.05 and 0.2 (respectively), statistical analysis calculated the required number of patients to be 30 patients. We evaluated OS and DFS by the Kaplan–Meier method, using SPSS software, version 22.0 (IBM Japan Ltd., Tokyo, Japan).
